# Prevalence and Causes of Hyperuricemia in Children

**DOI:** 10.7759/cureus.15307

**Published:** 2021-05-28

**Authors:** Jatender Kumar, Aarzoo Gupta, Kapeel Dev, Sameet Kumar, Deepak Kataria, Ambresha Gul, Mohammed Abbas, Amna Jamil, Simra Shahid, Sidra Memon

**Affiliations:** 1 Internal Medicine, Jinnah Postgraduate Medical Centre, Karachi, PAK; 2 Internal Medicine, Vardhman Mahavir Medical College and Safdarjung Hospital, Faridabad, IND; 3 Internal Medicine, Ghulam Muhammad Mahar Medical College, Sukkur, PAK; 4 Internal Medicine, Chandka Medical College, Larkana, PAK; 5 Internal Medicine, Shaheed Mohtarma Benazir Bhutto Medical University, Larkana, PAK; 6 Internal Medicine, Peoples University of Medical and Health Sciences for Women, Nawabshah, PAK; 7 Medicine, University of Louisville, Louisville, USA; 8 Medicine, Jinnah Sindh Medical University, Karachi, PAK; 9 Internal Medicine, Jinnah Sindh Medical University, Karachi, PAK

**Keywords:** hyperuricemia, uric acid, children, pediatrics, risk factors

## Abstract

Introduction

There are various factors responsible for hyperuricemia in children, however, there is extremely limited local data available. In this study, we aim to determine the causes and risk factors associated with hyperuricemia. This study will assist pediatric consultants to identify children who might be at risk of hyperuricemia and manage them accordingly.

Methods

This study was conducted in pediatric outpatient departments of various tertiary care hospitals from January 2018 to December 2019. Five thousand (n = 5000) children of either gender between the age group of 1-14 years, were enrolled in the study after informed consent from their parents. Uric acid levels were assessed by using the UASure blood uric acid monitoring handheld device.

Results

In our study, n = 1301 (26.02%) children had hyperuricemia. Hyperuricemia was more common in male compared to females (65.49% vs. 34.51%; p-value <0.00001) and in older children (9 ± 2 years vs. 7 ± 3; p-value <0.00001). In hyperuricemia patients, the most common disorder was gastroenteritis (23.98%), followed by respiratory infection (23.14%) and asthma (15.45%).

Conclusion

Hyperuricemia in children is very prevalent in the local setting. Patients with pre-existing conditions like congenital heart disease, asthma, epilepsy, and cancers should routinely be screened for hyperuricemia and managed accordingly to avoid long-term complications associated with hyperuricemia.

## Introduction

Hyperuricemia is a laboratory abnormality [1]. Considering its solubility, serum uric acid of greater than 7.0 mg/dL, is defined as hyperuricemia in adults [2]. But the important information that needs to be taken into consideration when defining hyperuricemia in the pediatric population is that uric acid levels change during development, increasing gradually from birth to the end of adolescence, sharply in males and slightly in females. Therefore, age and sex-related reference values for uric acid should be considered for defining hyperuricemia in children [3].

Some acute or chronic conditions that can be a major causative factor of increased uric acid in children and adolescents are Down’s syndrome, congenital heart diseases, metabolic or genetic diseases, gastroenteritis, bronchial asthma, and malignant disorders. Obesity is one of the major causes of hyperuricemia because of its association with metabolic disorders and non-communicable diseases like hypertension, insulin resistance, dyslipidemia, and chronic kidney disease [1]. Literature has reported high prevalence in the obese population than the general population and proved a direct association of increased BMI with elevated uric acid [4]. Another study in China has shown a high prevalence of hyperuricemia in children of age 3-6 years, male gender, and a strong association with high diastolic blood pressure (DBP) and increased triglycerides concentration [5]. Hyperuricemia can occur as a side effect of several drugs like some antiepileptics, with valproate and phenobarbital being the commonest ones; thiazide diuretics, cyclosporine, theophylline, and pyrazinamide have also been reported as the main causative factors, with their mechanism not fully understood [6].

Despite 35% of the population less than 14 years of age in Pakistan, there is extremely limited data available on causes and risk factors associated with hyperuricemia [7]. In this study, we aim to determine the causes and risk factors associated with hyperuricemia. This study will assist pediatric consultants to profile children who might be at risk of hyperuricemia and manage them accordingly.

## Materials and methods

This study was conducted in the pediatric outpatient departments of various tertiary care hospitals from January 2018 to December 2019. Five thousand (n= 5000) children of either gender between the age group of 1 to 14 years, were enrolled in the study after informed consent from their parents. Children older than five years were explained the procedure and their ascent was taken. Uric acid levels were assessed by using the UASure blood uric acid monitoring handheld device. Participants were labeled with hyperuricemia based on Mayo Clinic Laboratories reference value (Table 1) [8].

**Table 1:** Mayo Clinic Laboratories reference value for hyperuricemia.

Age	Reference Value
Male	
1-10 years	2.4–5.4 mg/dL
11 years	2.7–5.9 mg/dL
12 years	3.1–6.4 mg/dL
13 years	3.4–6.9 mg/dL
14 years	3.7–7.4 mg/dL
Female	
1 year	2.1–4.9 mg/dL
2 years	2.1–5.0 mg/dL
3 years	2.2–5.1 mg/dL
4 years	2.3–5.2 mg/dL
5 years	2.3–5.3 mg/dL
6 years	2.3–5.4 mg/dL
7-8 years	2.3–5.5 mg/dL
9-10 years	2.3–5.7 mg/dL
11 years	2.3–5.8 mg/dL
12 years	2.3–5.9 mg/dL
≥13 years	3.7–7.4 mg/dL

After testing for uric acid, participants were labeled as either a case or control group based on the presence or absence of hyperuricemia. Patient’s characteristics such as age, gender, and detailed history included the presence of active respiratory or gastrointestinal infection, pre-existing conditions like asthma, congenital heart disease, epilepsy, and the nephrotic syndrome were noted in a self-structured questionnaire. Participant’s BMI and blood pressure (BP) were checked and noted in a self-structured questionnaire.

Statistical analysis was done using the Statistical Package for the Social Sciences (SPSS) version 22.0 (IBM Corporation, Armonk, New York, United States). Continuous variables were analyzed via descriptive statistics and were presented as mean and standard deviation (SD) while categorical variables were presented by percentages and frequencies. Chi-square and t-test were used as appropriate. A p-value of less than 0.05 meant that there is a difference between the two groups and the null hypothesis is void.

## Results

In our study, n = 1301 (26.02%) children had hyperuricemia. Hyperuricemia was more common in males compared to their female counterparts (65.49% vs. 34.51%; p-value <0.00001) and in older children (9 ± 2 years vs. 7 ± 3; p-value <0.00001). Participants with hyperuricemia also had significantly higher BMI, systolic blood pressure (SBP), and DBP (Table 2).

**Table 2:** Characteristic of participants with hyperuricemia. DBP: Diastolic blood pressure; SBP: Systolic blood pressure.

Characteristics	Hyperuricemia (n = 1301)	Normouricemia (n = 3699)	P-value
Age in years (Mean ± SD)	9 ± 2	7 ± 3	<0.0001
Male (%)	852 (65.49%)	1871 (50.59%)	<0.00001
BMI (Mean ± SD)	19.2 ± 2.1	16.9 ± 1.8	<0.00001
SBP (Mean ± SD)	106.1 ± 6.2	99.5 ± 4.9	<0.00001
DBP (Mean ± SD)	68. 1 ± 3.6	62.1 ± 3.1	<0.00001
Breastfed	612	1802	0.29

In hyperuricemic patients, the most common disorder was gastroenteritis (23.98%), followed by respiratory infection (23.14%) and asthma (15.45%). Other prominent disorders were nephrotic syndrome (5.38%) and epilepsy (2.38%) (Table 3).

**Table 3:** Associated disorders with hyperuricemia. *Pneumonia, bronchitis, and respiratory syncytial infection. **Ventricular septal defect, atrial septal defect, tetralogy of Fallot, patent foramen ovale, and transposition of great arteries.

Disorders	Hyperuricemia (n = 1301)	Normouricemia (n = 3699)	P-value
Gastroenteritis	312 (23.98%)	306 (8.27%)	<0.00001
Respiratory infections*	301 (23.14%)	372 (10.06%)	<0.00001
Asthma	201 (15.45%)	281 (7.60%)	<0.00001
Nephrotic syndrome	70 (5.38%)	73 (1.97%)	<0.00001
Epilepsy	31 (2.38%)	19 (0.51%)	<0.00001
Congenital heart disease**	24 (1.84%)	27 (0.73%)	0.0005
Malignancy	19 (1.46%)	21 (0.57%)	0.0034
Down syndrome	17 (1.31%)	3 (0.08%)	<0.00001

## Discussion

Our study demonstrated that the prevalence of hyperuricemia in children, coming to hospital due to any ailment, was 26%. In another study, the prevalence of hyperuricemia in the adult population in Pakistan was 30.0% [9]. In our study, hyperuricemia was more common in males and older children. Moreover, SBP, DBP, and BMI were higher in participants with hyperuricemia. In addition to this, some of the disorders more common in hyperuricemic participants were gastroenteritis (23.98%), respiratory infections (23.14%), and asthma (15.45%). Among the prominent disorders, epilepsy and nephrotic syndrome were more common.

Several global studies have been conducted which are in line with this study. The incidence of gout was more common in males as compared to females [10]. Similarly, the study conducted by Kubota et al showed the presence of at least one underlying disorder among hyperuricemic patients [1]. Several factors have been identified which play a major role in causing hyperuricemia. Abnormalities during infancy or childhood in the metabolism of purine compounds have been shown to cause hyperuricemia [10]. Muscle exertion during glycogen storage diseases enhances the degradation of muscle purine nucleotides [11]. Moreover, Down syndrome and congenital heart diseases have also shown an association with hyperuricemia [12,13]. On the other hand, some of the acute disorders, including bronchial asthma, gastroenteritis, hemolytic anemia, and malignant disorders, also play an important role in causing hyperuricemia [14,15]. Feig and Johnson evaluated the positive correlation between the levels of uric acid, SBP, and DBP [16]. Similarly, our study also showed a positive correlation between them. In concordance with our study, hyperuricemia is considered to be an important risk factor for the development of diseases such as obesity, hyperlipidemia, and diabetes [17,18]. Hyperuricemia is a target of treatment as it increases the risk of mortality, particularly due to renal and cardiovascular diseases [19].

Since obesity is a major risk factor for hyperuricemia, lifestyle interventions including dietary modifications, physical activity, and behavioral changes to reduce weight are important. Moreover, xanthine oxidase inhibitors play an important role in treating several pediatric diseases. In addition to this, either used alone or in combination with enalapril, allopurinol helps in reducing BP in children with hyperuricemic hypertension [20]. On the other hand, uric acid oxidase (Rasburicase) results in a rapid decline in uric acid, compared to allopurinol [21]. However, these drugs should be used with caution, as they can cause severe skin side effects, including Stevens-Johnson syndrome, and hypersensitivity, etc. [22,23].

To the best of our knowledge, this is the first large-scale study that determines the prevalence of hyperuricemia in children in developing countries. Since the study was multicentric, our sample was diverse. However, a definite association between various diseases and hyperuricemia could not be established as the study was cross-sectional in nature. Another limitation is that the sample size was not taken from the general public, but from children coming to the outpatient department for various ailments, so care should be taken while inferring the calculated prevalence of hyperuricemia in the pediatric population to general population.

## Conclusions

This study reflects the contribution of the various causes such as age, obesity, infections, and malignancy in causing hyperuricemia. These factors are particularly important concerning chronic diseases that cause hyperuricemia, starting early in childhood. There should be a more thorough investigation, and the treatment should be streamlined accordingly. Moreover, if diagnosed earlier, it results in improvement. Therefore, pediatricians should pay greater attention to hyperuricemia in clinical settings.
